# From Water for Water: PEDOT:PSS-Chitosan Beads for Sustainable Dyes Adsorption

**DOI:** 10.3390/gels10010037

**Published:** 2023-12-31

**Authors:** Irene Vassalini, Marina Maddaloni, Mattia Depedro, Alice De Villi, Matteo Ferroni, Ivano Alessandri

**Affiliations:** 1 Sustainable Chemistry and Materials Laboratory, Department of Information Engineering, University of Brescia, Via Branze 38, 25123 Brescia, Italy; 2 Consorzio Interuniversitario Nazionale per la Scienza e Tecnologia dei Materiali (INSTM), Research Unit of Brescia, Via Branze 38, 25123 Brescia, Italy; 3 CNR-INO, Research Unit of Brescia, Via Branze 38, 25123 Brescia, Italy; 4 Chemistry for Technology Laboratory, Department of Mechanical and Industrial Engineering, University of Brescia, Via Branze 38, 25123 Brescia, Italy; 5 Department of Civil, Environmental, Architectural Engineering and Mathematics, University of Brescia, Via Branze 43, 25123 Brescia, Italy; 6 CNR-IMM Bologna, Via Gobetti 101, 40129 Bologna, Italy

**Keywords:** chitosan, environmental remediation, hydrogels, water pollutants, dye removal, food-waste materials

## Abstract

This study investigates the viability of developing chitosan-based hydrogels derived from waste shrimp shells for the removal of methylene blue and methyl orange, thereby transforming food waste into advanced materials for environmental remediation. Despite chitosan-based adsorbents being conventionally considered ideal for the removal of negative pollutants, through targeted functionalization with poly(3,4-ethylenedioxythiophene): polystyrene sulfonate (PEDOT:PSS) at varying concentrations, we successfully enhance the hydrogels’ efficacy in also adsorbing positively charged adsorbates. Specifically, the incorporation of PEDOT:PSS at a concentration of 10% *v*/*v* emerges as a critical factor in facilitating the robust adsorption of dyes. In the case of the anionic dye methyl orange (MO, 10^−5^ M), the percentage of removed dye passed from 47% (for beads made of only chitosan) to 66% (for beads made of chitosan-PEDOT:PSS 10%), while, in the case of the cationic dye methylene blue (MB, 10^−5^ M), the percentage of removed dye passed from 52 to 100%. At the basis of this enhancement, there is an adsorption mechanism resulting from the interplay between electrostatic forces and π–π interactions. Furthermore, the synthesized functionalized hydrogels exhibit remarkable stability and reusability (at least five consecutive cycles) in the case of MB, paving the way for the development of cost-effective and sustainable adsorbents. This study highlights the potential of repurposing waste materials for environmental benefits, introducing an innovative approach to address the challenges regarding water pollution.

## 1. Introduction

The escalating global population demands innovative strategies to secure clean water access and promote sustainable development [1]. On one hand, there are severe problems linked to hygiene and sanitization, especially in the poorest regions of the world; on the other hand, the presence of different kinds of pollutants significantly lowers the quality of water, even in most developed countries. Particularly relevant are organic dyes, which are extensively used in different industrial sectors: textile, tannery, paper and pulp industries, or dye manufacturing. Recent studies reported that, annually, about 7 × 10^7^ tons of synthetic dyes are produced worldwide [2], and the global dyes market and dyeing industry are expected to grow in the next few years [3]. Unfortunately, the concentration of organic dyes in textile wastewater can be as high as 250 mg/L [4], posing a serious hazard to the environment. Even at low concentrations, they can promote toxicity, mutagenicity, and carcinogenicity, and they enter the food chain, providing recalcitrance, bioaccumulation, and secondary pollution. In addition, they can cause skin irritation, allergies, or gastrointestinal and respiratory diseases [2]. Despite their chemical variability, all synthetic dyes share common characteristics: high solubility in water and exceptional stability under solar light and environmental oxidation. For these reasons, conventional wastewater treatment options are frequently ineffective, and innovative advanced decontamination approaches are needed. Different removal strategies have been proposed, including coagulation, flocculation, advanced oxidation, ion-exchange, electrochemical treatments, and biological degradation [3], and adsorption (a surface-based process in which adsorbed molecules or ions are attracted to a solid adsorbent surface) is often considered a viable option. Activated carbons (AC) are the commercial standard for adsorption systems for the treatment of industrial wastewater, but their production and regeneration are characterized by high costs and require treatment at very high temperatures. In addition, they derive from fossil fuels, imposing limitations and doubts regarding their extended use. On the contrary, the preparation and employment of the adsorption system should be characterized by a low environmental impact: the use of natural materials and their processing according to sustainable chemical processes, could be a viable virtuous strategy [5]. From this perspective, the possibility of utilizing food waste as a starting point for the extraction of chemicals to prepare high-added-value decontaminant systems is particularly appealing [6].

Recent studies have demonstrated that the amount of household food waste produced is as high as 60–110 kg/capita/year, according to world localization [7]. The extraction of valuable chemicals and raw materials from food waste has been widely exploited in the fields of packaging [8,9,10], cosmetics [11], pharmaceutics [12,13,14,15,16], and green electronics [17], and, recently, it has started to be extended to the field of environmental remediation [18].

Unfortunately, nowadays, only 1% of food waste is recycled for industrial uses [19], and application in environmental remediation deserves further investigation and development. The idea at the basis of this work is that of extracting chitin from discarded shrimp shells [20], converting it into chitosan by deacetylation, and creating hydrogel beads that can be used as adsorbents of dyes from wastewater. This is a win–win approach: on one hand, what is considered waste is converted into a high-added-value product; on the other, water quality can be improved. Other advantages are tightly linked to the proposed system: chitosan is a biocompatible and biodegradable natural polymer, and the preparation of the adsorbent system in the form of beads is extremely simple and guarantees the adsorbent removal just by using a common filtering system or tweezers. In addition, chitosan is characterized by antibacterial activity, which can be exploited to further enhance water quality [21]. This last aspect represents a significant advantage in comparison to other natural biopolymers, such as pectin and alginate, from which similar hydrogel-based adsorbents can be derived [22,23]. However, chitosan maintains the possibility to incorporate different functional units inside the hydrogel systems, enhancing/modifying their functional properties. Chitosan is commonly considered a positively charged polymer under standard conditions (at almost neutral pH) since its amine units are protonated (pK_a_~6.2–6.5 [24,25]), and this property makes it ideal for interaction and adsorption of negatively charged molecules [26,27,28,29,30].

Our aim was to create a chitosan-based adsorbent also efficient in the adsorption of negative molecules. To pursue this scope, we incorporated an additional functional unit into the chitosan matrix, enhancing its interaction with synthetic dyes thanks to the presence of an extended structure of conjugated π bonds.

In particular, we inserted inside the hydrogel matrix a derivative of polythiophene, the poly(3,4-ethylenedioxythiophene): polystyrene sulfonate (PEDOT:PSS), which is one of the most investigated conductive polymers in the field of large-area flexible electronics because of its biocompatibility, chemical stability, and electrical properties.

Recently, it has been employed also for environmental remediation and dye removal, but, up to now, PEDOT has been mainly used as a co-catalyst to enhance the photo-degradation activity of some semiconductors [31,32,33,34]. Its use as an adsorbent has been only marginally investigated and deserves further study. Da Silva et al. [35] showed the possibility of using PEDOT in combination with polyvinylidene fluoride (PVDF) mats, which served as mechanical supports, for the adsorption of methyl orange (MO). In this case, the interaction was mainly based on electrostatic forces between the positive surface charge of the adsorbent (obtained at pH < 5) and the negative charge of the dye. Due to the electrostatic nature of the interaction, the proposed system was quite specific towards MO or analogous negative dyes, and its activity was limited at strongly acidic pH.

In this work, we aimed to develop a more universal adsorbent: the exploitation of π interaction between the conjugated system of PEDOT and the benzene units of synthetic organic dyes could be a successful and innovative strategy to remove synthetic dyes independently of their charge. In addition, PEDOT is generally commercially available in formulation with polystyrene sulfonate (PSS), which is negatively charged, guaranteeing additional electrostatic interaction with positively charged pollutants.

MO and methylene blue (MB) were selected as representatives of negatively and positively charged dyes, respectively. Their aqueous solutions were treated with hydrogel beads made of pure chitosan (named “only ch” beads) and chitosan + PEDOT:PSS (named “ch-PEDOT:PSS” beads), and the amount of adsorbed dye as a function of time was measured until reaching the equilibrium of the adsorption process. The influence of different experimental parameters on the adsorption performances was investigated, such as the value of the external pH, the amount of PEDOT:PSS introduced inside the hydrogel beads (0.1–10% *v*/*v*), or the co-presence of more dyes inside the same solution. Furthermore, we tried to obtain insights into the mechanism at the basis of the adsorption process and tested the possibility of reusing the same adsorbent system for successive treatments of different dye solutions.

## 2. Results and Discussion

**Figure 1:**
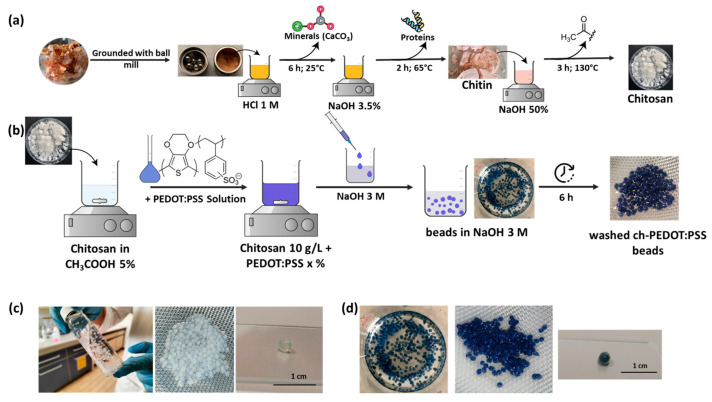
Scheme of the synthesis process of chitosan or PEDOT:PSS-chitosan beads. (**a**) Extraction of chitin and chitosan from shrimp shells; (**b**) sol–gel synthesis of (PEDOT:PSS-)chitosan beads; (**c**) optical images ofchitosan beads; (**d**) optical images of ch- PEDOT:PSS (5% *v*/*v*) beads.

Household food wastes in the form of shrimp shells and heads were treated with HCl and NaOH and converted into chitosan, following the process reported in [36] and in the Materials and Methods section and schematized in Figure 1a. To verify the demineralization of the crustacean shells and deacetylation processes of the obtained chitin, the final powder was characterized by means of IR spectroscopy. As illustrated in Figure S1, the spectrum exhibits all the characteristic peaks of the main functional groups typical of chitosan: –NH_2_ and –OH at 3413 cm^−1^, –C–H at 2919 cm^−1^, –CO at 1654 cm^−1^, –CH_2_ at 1377 cm^−1^, and –C–O–C at 1022 cm^−1^, confirming the chemical conversion of the initial food waste. Deacetylation of chitin into chitosan was also confirmed through Raman spectroscopy: Figure S2 shows the typical increase in intensity of the peak centered at 2885 cm^−1^ (attributed to CH_2_ stretching) in comparison to the peak at 2919 cm^−1^ (attributed to CH_3_ stretching), along with the increase in the intensity of the peak at 896 cm^−1^ (associated with stretching and rocking modes of CH_2_ (CH_2_ν(φ) + ρCH_2_)) in comparison to the peak at 936 cm^−1^ (due to CN stretching) [37].

The obtained powder also underwent titration with NaOH to determine the degree of deacetylation, which is a fundamental parameter for the creation of stable physical chitosan hydrogel. One of the simplest ways to obtain physical hydrogel from chitosan is by playing with its different solubility as a function of pH: chitosan is highly soluble in water at acidic pH, but it tends to precipitate in an alkaline environment. Consequently, it is possible to jellify its aqueous solutions without the addition of any crosslinker by direct exposure to an alkali medium under well-defined conditions. In alkaline conditions, hydrophobization of the polymer chains occurred, leading to their condensation to form a three-dimensional network, which resulted in a dense gel. However, the existing literature indicates that stable physical hydrogels can only be obtained starting from highly deacetylated chitosan [38]. As reported in Section S3 of Supplementary Materials, the obtained chitosan was characterized by a deacetylation degree of ~80%, which is ideal for the creation of stable pure chitosan hydrogels [38].

Following the procedure reported in the Materials and Methods section and schematized in Figure 1b, it was possible to produce chitosan-based hydrogels containing a variable amount of PEDOT:PSS (from 0.1 to 10% *v*/*v*) characterized by a spherical shape with a diameter in the order of few millimeters (~3 mm). The final shape and dimensions strongly depended on the pressure applied to the syringe piston, the dimensions of the syringe needle, and the fall distance of the chitosan acidic solution into the alkaline medium used for jellification. In addition, the inclusion of PEDOT:PSS in the chitosan acidic solution modified its viscosity and influenced the final shape and size of the produced beads. In general, PEDOT:PSS-chitosan beads with a high content of PEDOT:PSS were characterized by smaller dimensions (Figure 1c,d). Another obvious distinction between chitosan beads containing or not-containing PEDOT:PSS was the color as a higher content of PEDOT:PSS corresponded to a darker color. The inclusion of PEDOT:PSS inside the chitosan matrix was also verified by means of Raman spectroscopy. The Raman spectra reported in Figure S4a show that beads made of only chitosan lacked significant peaks, while chitosan beads with embedded PEDOT:PSS (10% *v*/*v*) clearly exhibited the main peaks proper of PEDOT:PSS: at 1260 cm^−1^ the band corresponding to C_α_–C_α′_ inter-ring stretching between thiophene units; at 1370 cm^−1^ the peak due to C_β_–C_β_ and C_α_–C_α′_ stretching and ν_2_ mode (intra-ring –C_3_–C_4_– and –C_4_–C_5_– in-phase stretching coupled with –CH bending); at about 1430 cm^−1^ the main band due to the symmetric stretching of the C_α_–C_β_ bonds of thiophene rings and the peak due to the asymmetric stretching of the C_α_–C_β_ bonds of thiophene rings, together with the ν_1_ mode [39]. The typical spectroscopic features of chitosan, instead, are clearly visible in the FTIR spectra of both only chitosan and ch-PEDOT:PSS 10% dried beads (Figure S4b).

The morphology and internal structure of the synthesized beads were also investigated through ESEM (Environmental Scanning Electron Microscope), which enabled to study the beads before complete drying. The analysis demonstrated the presence of a highly interconnected polymeric structure and internal porosity, evident in both PEDOT:PSS functionalized and not-functionalized chitosan beads (Figure S5).

Methylene blue (MB, 10^−5^ M) and methyl orange (MO, 10^−5^ M) were selected as examples of cationic and anionic dyes, respectively. Initially, the adsorption efficiency of different chitosan-based beads, containing variable amounts of PEDOT:PSS, was evaluated in not-buffered (natural) conditions. Due to variations in the sizes and weights of the produced beads, a fair comparison among the different tested systems was ensured by keeping the number of beads in contact with the dye solution constant. The weight of the beads was used to normalize the amount of adsorbed dye and calculate the corresponding adsorption capacity, q (mg/g). Specifically, the change in adsorption capacity over the soaking time of the beads (*q_t_*) was evaluated until it reached adsorption equilibrium after 21.5 h. Noteworthy is the fact that the here-reported values of *q_t_* were calculated considering the weight of the beads in their hydrated form: since the water content inside the hydrogels was ~98% of their weight (i.e., 98.65% for only chitosan beads, and 97.92% for ch-PEDOT:PSS 10% beads), this fact (together with the lower starting concentration of MB and MO) intrinsically led to *q_t_* values lower than those commonly reported in the literature [30,40,41,42].

**Figure 2:**
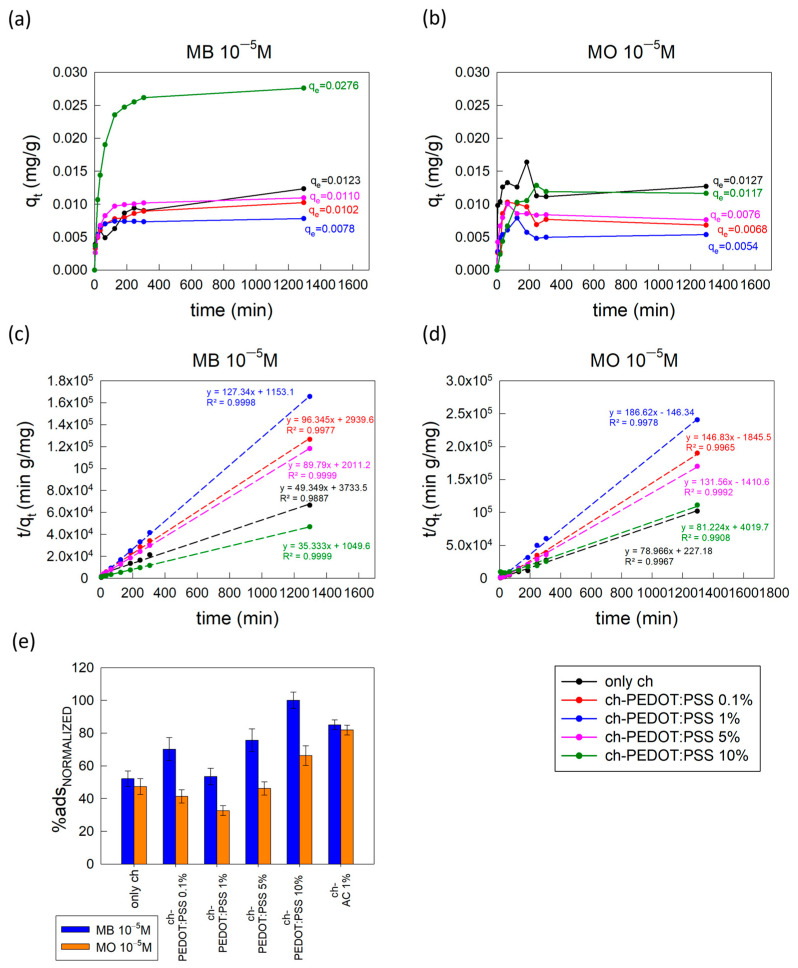
Comparison between the adsorption behavior of different types of chitosan-based beads during the interaction with MB and MO 10^−5^ M solutions. (**a**) Variation in the adsorption capacity of pure chitosan beads and chitosan beads functionalized with PEDOT:PSS (0.1–10% V) as a function of beads’ soaking time inside an MB 10^−5^ M solution; (**b**) variation in the adsorption capacity of pure chitosan beads and chitosan beads functionalized with PEDOT:PSS (0.1–10% V) as a function of beads’ soaking time inside a MO 10^−5^ M solution; (**c**) pseudo-second-order kinetic model for adsorption of MB with different chitosan and chitosan-PEDOT:PSS beads; (**d**) pseudo-second-order kinetic model for adsorption of MO with different chitosan and chitosan-PEDOT:PSS beads; (**e**) variation in the normalized adsorption percentage of MB and MO according to chemical composition of chitosan-based beads. When error bars are not shown, standard deviation is included in dots’ size.

All the tested systems were characterized by a pseudo-secondo order kinetic behavior: as visible in Figure 2a,b, fast adsorption occurred in the first period, followed by a decrease in the adsorption rate, until the reaching of a plateau value typical of the reaching of equilibrium. We performed the fitting of the experimental data according to the linearized form of different adsorption kinetic models (pseudo-first-order, pseudo-second-order, liquid-film diffusion, intraparticle diffusion, and Elovich models), and the obtained results are reported in Figure S6 (for MB) and Figure S7 (for MO). In the case of both dyes, all the experimental data could be satisfactorily fitted according to the linearized form of the pseudo-second-order model (Figure 2c,d). It was possible to calculate the theoretical values of the equilibrium adsorption capacity, showing a good agreement with the experimental data (Table S1).

Pure chitosan beads resulted to be a discrete adsorbent system both for MO (% adsorption = 47.3%, *q_e_* = 0.0127 mg/g) and MB (% adsorption = 52.13%, *q_e_* = 0.0123 mg/g), without showing a clear preference/specificity for positively or negatively charged dyes. The reason could be linked to the fact that, under natural conditions (i.e., without any external pH buffering), the pH of the dye solutions was equal to 6.2 and the chitosan chains were only slightly protonated (chitosan pk_a_ ~6.5 [24]). As a result, the electrostatic attraction with negatively charged species in solution (i.e., MO^−^) was limited, as well as the electrostatic repulsion towards positively charged species (i.e., MB^+^). In addition, since chitosan crosslinking was performed through reaction with NaOH, some hydroxyl groups can remain entrapped inside the hydrogel matrix, compensating positive charges on the polymeric network and promoting the interaction with positive MB^+^ species in solution.

The incorporation of PEDOT:PSS inside the chitosan hydrogel matrix modified the adsorption performances of the beads. To quantify the effect of the conductive polymer, we calculated the normalized adsorption percentage. Pure chitosan beads, corresponding to the not-functionalized adsorbent, were considered a sort of reference material and their weight was used to normalize the amount of all the other adsorbent systems. The obtained data are plotted in Figure 2e, which illustrates that functionalization led to different behaviors in the case of MO and MB.

When the positively charged MB was considered, the following general trend was observed: the normalized adsorption percentage increased when the amount of PEDOT:PSS raised (with the only exception of beads containing PEDOT:PSS at 1% *v*/*v*). When the concentration of PEDOT:PSS was low (0.1–1%), the adsorption was very similar or only slightly enhanced in comparison to pure chitosan beads, while the enhancement became significant when the content of PEDOT:PSS exceeded 5%.The reasons at the basis of this behavior could be manifold: on one hand, polystyrene sulfonate units (pka~1) at the considered not-buffered pH (~6.2) are negatively charged; they can favor an electrostatic interaction with MB^+^ ions in solution and foster its adsorption; on the other hand, PEDOT and PSS are characterized by an extended conjugated system that can enable not-electrostatic π–π interactions with the aromatic moieties of the organic dye [33].

A similar approach was proposed by Mittal et al. [43], by incorporating graphene oxide (GO) inside hydrogel nanocomposites made of chitosan/carboxymethyl cellulose, which demonstrated to be capable of satisfactorily adsorbing both MB and MO. However, the authors ascribed the high efficiency in MB adsorption not only to π–π interactions between the dye and GO but also to electrostatic interactions between the dye and negatively charged carboxymethyl cellulose.

The importance of the not-electrostatic interactions became evident by comparing the adsorption performances of ch-PEDOT:PSS 10% and only chitosan beads with that of chitosan beads containing only PSS (in the same amount of ch-PEDOT:PSS 10% beads), as reported in Figure S8a. Ch-PSS beads, in fact, were characterized by an MB-normalized adsorption higher than only chitosan beads but lower than ch-PEDOT:PSS 10% beads.

**Scheme 1:**
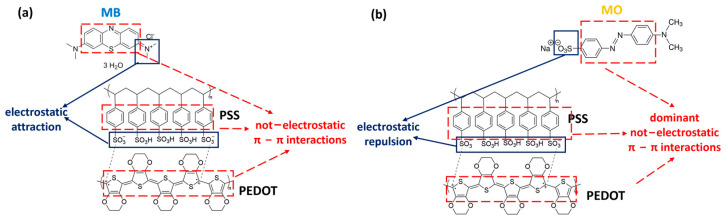
Possible interactions between PEDOT:PSS and organic dyes in not-buffered conditions. (**a**) The case of methylene blue; (**b**) the case of methyl orange.

Furthermore, PEDOT at the considered pH should not be positively charged, limiting any electrostatic repulsion with MB^+^ [32] (Scheme 1a).

In the case of negatively charged MO (pka~3.39), a variable trend was observed, as reported in Figure 2e. When the content of PEDOT:PSS was low, in the order of 0.1–5%, a reduction in dye adsorption was observed in comparison to pure chitosan beads. Nevertheless, the MO adsorption was not zero thanks to the interaction with the chitosan matrix. Surprisingly, when the PEDOT:PSS concentration was increased to 10%, a significant increase in adsorption was observed in comparison to pure chitosan beads, indicating that, in these optimal conditions, the contribution of not-electrostatic forces based on π–π interactions became prevalent (Scheme 1b). Anyway, the enhancement of adsorption was significantly more contained in comparison to MB.

Again, the not-electrostatic interactions became evident in the case of ch-PSS beads, which were characterized by surprisingly high adsorption percentage (the normalized adsorption percentage resulted in being equal to 100%, as visible in Figure S8b). Despite both PSS and MO being negatively charged, ch-PSS beads were excellent adsorbers for MO. In these regards, it has to be underlined that part of this increased adsorption could be linked to morphological considerations: the ch-PSS beads were significantly smaller than the other prepared beads (their hydrated weight was about half of the hydrated weight of ch-PEDOT:PSS 10% beads), leading to higher values of specific surface area. Furthermore, they were significantly less stable since they tended to dissolve and formed small pieces of gels inside the dye solution, increasing the surface area available for adsorption processes. This instability could derive from the fact that the PSS used for beads preparation was in the form of sodium salt and Na^+^ ions could interact with hydroxyl ions used for beads gelation and crosslinking, reducing their durability. Despite this fact being an evident drawback in practical applications, the formation of small gel fragments can lead to an increase in exposed adsorption sites and a consequent rise in dye removal.

Interestingly, from Figure 2e, it is also clear that the adsorption performances against MB of chitosan beads containing PEDOT:PSS at 10% *v*/*v* were also higher than those of similar chitosan beads containing activated carbons (ch-AC-1% *v*/*v* beads). The concentration of AC inside chitosan beads was lower than PEDOT:PSS, but it was selected because higher AC content led to unstable beads. For this type of system, any electrostatic interaction was not expected, as confirmed by the very similar values of normalized adsorption percentage obtained in the case of MB (85%) and MO (82%).

Although the creation of a not-specific adsorbent could be desirable, especially in view of application in real water systems characterized by the simultaneous presence of different and differently charged pollutants, we tried to modulate the adsorption capacity of the ch-PEDOT:PSS-tested systems by modifying the solution pH. In this way, it was possible to gain insights into the adsorption mechanism and identify the best conditions to maximize the adsorption process for each organic dye. For this scope, we considered two different pH values in addition to the natural one of the dye solutions (pH = 6.2): an alkaline pH of 9.8–9.9, and a more acidic value of 5.6. Unfortunately, it was not possible to work in more acidic conditions, reaching values of pH that were lower than the pK_a_ of MO (3.39, to protonate it and nullify the negative charge on the sulfonate group) or that enable the creation of polarons or bipolarons in PEDOT units (which originate at pH < 5.2) to introduce some additional positive charges inside the beads. In fact, in those strongly acidic conditions, the beads underwent progressive dissolution.

**Figure 3:**
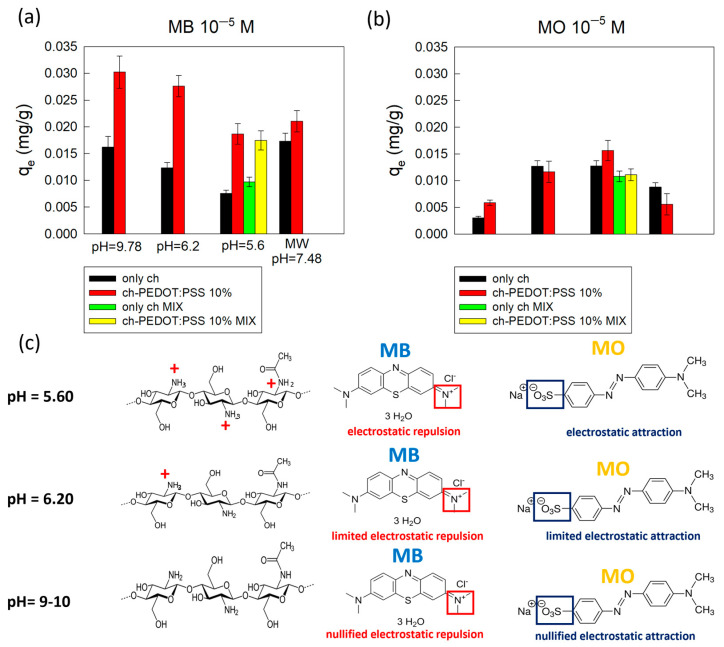
Variation in the equilibrium adsorption capacity (*q_e_*) of pure chitosan and chitosan-PEDOT:PSS (10%) beads as a function of solution pH, in the case of methylene blue 10^−5^ M (**a**) and methyl orange 10^−5^ M (**b**). (**c**) Proposed mechanism of interaction between chitosan and the two organic dyes at the base of the variation in adsorption performances as a function of pH.

For this analysis and optimization process, we restricted the investigation to pure chitosan beads and ch-PEDOT:PSS 10% since they were the most performing systems in not-buffered conditions. Inside the investigated pH range, all the tested beads maintained a second-order adsorption behavior, considering both MO and MB solutions, as shown in Figures S9 and S10, plotting the variation in the value of *q_t_* as a function of contact time. Interestingly, the value of adsorption equilibrium capacity *q_e_* significantly changed according to the pH value of the dye solution and according to the type of dye (positively or negatively charged). As visible in Figure 3a, in the case of MB, by increasing the pH value, it was possible to enhance the adsorption of the beads, especially in the case of pure chitosan. In fact, *q_e_* passed from 0.0076 mg/g at pH 5.6 to 0.0162 mg/g at pH 9.8, reaching a value that was more than double. This behavior could be explained considering that, in an alkaline medium, the positive charges localized on the amine groups of chitosan were nullified, limiting the electrostatic repulsion between them and positive MB^+^ in solution (Figure 3c). Furthermore, at this pH, the amine groups were in the unprotonated form, and nitrogen was bonded to three other atoms (alkyl group and two hydrogens) and had a lone pair of not-bonding electrons. This lone pair produced a region of electron density on the nitrogen atom, which, coupled with the electronegativity of nitrogen, favored the interaction with positively charged species in solution. The variation in *q_e_* had a similar trend also in the case of ch-PEDOT:PSS 10% beads (higher value of *q_e_* for alkaline pH), but it was less evident: *q_e_* passed from 0.0187 mg/g at pH 5.6 to 0.03 mg/g at pH 9.8, with a relative increase limited to 62%. This fact further demonstrated that, in the case of functionalization, electrostatic interactions between chitosan chains and dye in solution were less important but prevailed π–π interactions between PEDOT and the organic dye.

In the case of MO, an opposite behavior was observed: a decrease in adsorption capacity was recorded when the pH was increased. This effect held true for both pure chitosan beads and PEDOT:PSS-functionalized ones. In the first case, *q_e_* passed from 0.0127 mg/g at pH 5.2 to 0.0030 mg/g at pH 9.9, with a relative decrease of 75%, while, in the case of PEDOT-containing beads, *q_e_* decreased from 0.0153 mg/g at pH 5.2 to 0.0059 mg/g at pH 9.9, with a relative decrease of 62%. Also, for MO, the variation in *q_e_* as a function of pH could be explained by considering electrostatic forces at the basis of the interaction between the adsorbent and the dye. At high values of pH (9.9), MO was negatively charged, while chitosan was in its unprotonated form: through the lone pair of not-bonding electrons localized on the nitrogen atom, it repelled MO anions in solution, and adsorption was possible only through weak forces, such as Van der Waals or H-bonds (Figure 3c). Despite the additional repelling negative charges present in the case of functionalized beads due to polysulfonate units, higher adsorption was obtained at high pH (*q_e_* = 0.0059 mg/g versus 0.0030 mg/g) thanks to π–π interactions between PEDOT ions and aromatic rings of MO, as previously discussed and shown in Scheme 1b. At lower values of pH (6.2 and 5.6), instead, the number of positive charges on the chitosan polymeric network increased, favoring the interaction with MO^−^ in solution and increasing its adsorption. Between the two values of acidic pH, no differences in *q_e_* were observed since no significant variation in the chitosan polymeric chain from an electrostatic point of view occurred, only a slight variation in the number of positive charges on the chitosan chains. Furthermore, also in acidic conditions of pH 5.6, beads containing PEDOT:PSS outperformed pure chitosan beads, indicating the persistence of the contribution of π–π interactions (Scheme 1b).

In order to investigate the behavior of the synthesized beads in experimental conditions more similar to real-world conditions, we analyzed their adsorption capacities in acidic conditions when both the dyes were simultaneously contained inside the solution (adsorption curves reported in Figure S11). Interestingly, as reported in Figure 3a,b, the beads were able to efficiently adsorb both MB and MO without any significant reduction in *q_e_* in comparison to the values obtained when a single dye was contained in solution. If we consider the adsorption of MB in the presence of MO, for ch-PEDOT:PSS 10% beads, *q_e_* was lowered by only 6.5%, while, for pure chitosan beads, a slight increase of 28% was recorded in comparison to pure MB solution. It was evident that MO did not hinder MB adsorption. MB, instead, slightly lowered MO adsorption since *q_e_* was reduced by 15% in the case of pure chitosan beads and 27.5% in the case ch-PEDOT:PSS 10% beads. This behavior seems to be the opposite of that observed by Kang et al. [44] using as adsorbent exfoliated montmorillonite/chitosan gels (EMCG), but -it has to be underlined that EMCG systems were characterized by a negative surface charge, which can be “consumed” by MB, favoring MO adsorption. In the present study, instead, it is more probable that MB molecules saturated some aromatic adsorption sites of the hydrogels and limited their availability for π–π interactions with MO.

To prepare true-to-life samples, we dissolved MB and MO in mineral water (MW; see Table S2 for its chemical composition) to reach the same 10^−5^ M concentration and we tested pure chitosan and ch-PEDOT:PSS 10% beads. Also in this case, satisfactory adsorption was maintained in the case of pure chitosan beads, while PEDOT-containing beads showed a more environment-sensitive behavior (Figure 3a,b). In the case of MO, for pure chitosan beads, a value of *q_e_* intermediate to that measured at pH 9.9 and that of 6.2 was obtained, as expected considering that the pH of the mineral solution was 7.48. In the case of PEDOT-functionalized beads, instead, more limited adsorption was achieved, probably because of the interference from other ionic species contained in the solution. A similar trend was observed for MB. In the case of pure chitosan beads, the value of *q_e_* obtained in mineral water was increased in comparison to the solution prepared in Milli-Q water (solution with pH 6.2) thanks to the elimination of all the positive charges localized on the polymeric chain and the electrostatic repulsion with MB^+^ in solution. The increase in adsorption was even higher than that obtained in the case of alkaline solution prepared in Milli-Q water, indicating that, in this condition, ions contained inside the mineral water can favor MB^+^ adsorption. In the case of PEDOT-PSS containing beads, instead, a reduced value of *q_e_* was achieved (significantly lower than those measured both at pH 9.8 or 6.2), suggesting competition during adsorption between aqueous ions and dye molecules. This systematic reduction in the adsorption capacity in the presence of competitive chemical species observed for both the dyes, independently from their charge, was a further confirmation of the fact that PEDOT:PSS contribute to dye adsorption not only through electrostatic forces but also through π–π interactions.

**Figure 4:**
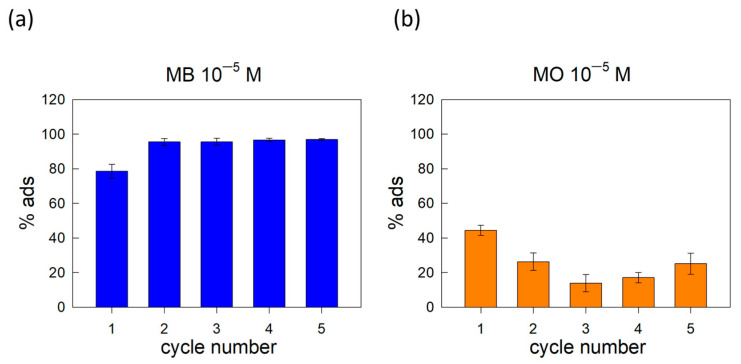
Reusability of chitosan-PEDOT:PSS 10% beads for MB and MO adsorption. (**a**) Variation in the % of MB 10^−5^ M adsorbed by hydrogel beads as a function of the number of consecutive adsorption experiments; (**b**) variation in the % of MO 10^−5^ M adsorbed by hydrogel beads as a function of the number of consecutive adsorption experiments.

Finally, we tested the possibility of reusing chitosan-PEDOT:PSS 10% beads for consecutive remediation experiments in not-buffered pH conditions, evaluating the stability of their adsorption performances. For these experiments, after a first adsorption test lasting 24 h, beads were removed from the dye solution and put in contact with a sodium dodecyl sulfate (SDS) 0.1 M solution for 24 h to extract the previously adsorbed dye. Subsequently, the same beads were washed with clean Milli-Q water and put again in contact with the dye solution. The same procedure was repeated to reach a total of five consecutive adsorption tests. The obtained results are summarized in Figure 4, where it is shown that, in the case of MB adsorption, ch-PEDOT:PSS 10% beads were characterized by very high reusability, outperforming similar chitosan-based adsorbents both in terms of easiness of the regeneration process and reduction in adsorption efficiency during the reuse [26,45]. In fact, the adsorption percentage of the successive tests was comparable to or even higher than the previous ones: if during the first cycle the percentage of MB 10^−5^ M adsorbed was equal to 78.5% (not-normalized value), it increased up to 95.5–96.9% during the second–fifth cycles. This adsorption enhancement could be justified considering that SDS is an anionic surfactant capable of fostering electrostatic interactions with MB. This peculiarity covered a fundamental role in the desorption of MB from beads after their first use, as well as in successive adsorption cycles. SDS, in fact, was used at a very high concentration, well above its critical micelle concentration (CMC = 8.25 × 10^−3^ M for pure SDS [46]) such that, inside the solution, multiple micelles were present and could favor MB desorption from the beads. Furthermore, even if after the desorption experiment chitosan-PEDOT:PSS 10% beads were thoroughly washed with water, some SDS and negative charges could remain attached on their surface and led to an empowered adsorption of positive species. Obviously, in the case of negative species, such as MO, an opposite behavior was observed. Even if SDS enabled to remove the previously adsorbed MO (release of ~80% of the MO adsorbed), the bead adsorption efficiency decreased in successive cycles, passing from 44% (not-normalized value) in the first cycle to ~25% in the fifth cycle, reaching a minimum in the third cycle (~14%). Again, the explanation of this behavior can be linked to the retention on the bead surface of negative charges deriving from SDS after the release experiment. In order to circumvent this limitation in beads’ reusability for MO removal, we tested a cationic surfactant (cetyltrimethylammonium bromide, CTAB) with a concentration above its CMC limit (the CMC value of pure CTAB is 1.0 mM, [47]). Unfortunately, we verified that, in these conditions, chitosan-PEDOT:PSS 10% beads were not stable but tended to dissolve in the external environment, probably because CTAB micelles favored the release of NaOH used as a crosslinking agent for the synthesis of the beads. Further studies will be performed to investigate this phenomenon and to find a way to increase the beads’ stability.

## 3. Conclusions

In this work, we investigated the possibility of preparing chitosan-based hydrogels for the removal of methylene blue (10^−5^ M) and methyl orange (10^−5^ M), starting from discarded shrimp shells, converting food wastes into advanced materials for environmental remediation. Conventionally, chitosan-based materials are considered ideal candidates for the adsorption of negative adsorbates, but the here-described hydrogels were characterized by good adsorption performances towards both cationic and anionic dyes: in not-buffered conditions, the removal of MB was equal to 52% and that of MO was equal to 47%. These performances were improved through pH control (increasing pH for MB and lowering pH for MO) and proper functionalization with PEDOT:PSS. The maximum effect was obtained when the content of PEDOT:PSS was equal to 10% *v*/*v*), which enabled to increase MO adsorption to 66% (in not-buffered conditions) and MB adsorption to 100%. At the basis of this enhanced adsorption, there was an interplay between different chemical/physical interactions (electrostatic interactions and π–π interactions). The prepared functionalized hydrogels were characterized by high stability and reusability (at least for 5 cycles), especially in the case of MB, paving the way to the development of efficient, cheap and sustainable adsorbents.

## 4. Materials and Methods

### 4.1. Extraction of Chitosan from Waste Shrimp Shells and Its Chemical Characterization

Chitosan has been obtained from shrimp shells. The extraction procedure is the same followed in Ref. [18]. Indeed, 10 g of raw materials (crustacean shells and heads) were washed, dried in an electric oven at 150 °C for 30 min, and finely ground by means of a ball mill (MM400 Retsch, Haan, Germany). The obtained powder was treated with 150 mL of HCl 1 M for 6 h at room temperature under magnetic stirring to achieve sample demineralization. The produced powder was washed with water until pH = 7 was reached, and then allowed to dry. Subsequently, deproteinization was obtained by adding 100 mL of NaOH (3.5%) to the powder and heating it mildly at 65 °C on a heating plate for 2 h. The obtained powder was chitin, which was washed with water until pH = 7 was reached, and then it was dried. Finally, chitosan was obtained by deacetylation, by treating the chitin powder with 50 mL of NaOH 50% and heating it on a magnetic plate at 130 °C for, at least, 3 h. In the end, the recovered sample was washed with water until pH = 7 was reached and it was dried.

The chemical structure of the produced powder was characterized by means of IR spectrometry (Equinox 55 spectrometer, Bruker, Billerica, MA, USA) and Raman spectroscopy (Labram HR-800 spectrophotometer (Horiba/Jobin Yvon, Kyoto, Japan) with a He−Ne laser source (λ = 632.8 nm), acquisition time 30 s).

The deacetylation degree of chitosan was calculated through potentiometric titration following the procedure reported in Ref. [48]: 0.2 g of dried chitosan powder was dissolved in 20 mL of HCl 0.1 M and 25 mL of Milli-Q water. After 30 min of continuous stirring on the heating plate to promote the dissolution, an additional 25 mL aliquot of Milli-Q water was added, and stirring continued for another 30 min. When the biopolymer was completely dissolved, the solution was titrated, adding NaOH 0.1 M until a constant pH of 11 was reached. The variation in pH was measured with a SI Analytics, Lab 845, pH meter. For calculation details, see Section S3 in Supplementary Materials.

### 4.2. Synthesis and Characterization of Chitosan and PEDOT-Chitosan Hydrogel Beads

Then, 0.10 g of chitosan was dissolved in 10 mL of 5% (*v*/*v*) acetic acid to obtain a 10 g/L solution. The dissolution process was accelerated thanks to the use of an ultrasonic bath or magnetic stirring. The prepared solution was then poured dropwise by a syringe in a solution of 3 M NaOH used as a crosslinker. The crosslinking step lasted 6 h. Subsequently, the obtained beads were recovered and washed with Milli-Q water until a neutral pH was reached.

The same synthetic steps were followed in the case of ch-PEDOT:PSS beads, with the only difference being the composition of the starting acidic chitosan solution. In fact, maintaining fixed the chitosan and the acetic acid concentrations (10 g/L and 5% *v*/*v*, respectively), variable amounts of PEDOT:PSS commercial solution (Sigma Aldrich, St. Louis, MO, USA) were added to reach a concentration of 0.1%, 1%, 5%, or 10% *v*/*v*. The obtained beads were called ch-PEDOT:PSS 0.1%, ch-PEDOT:PSS 1%, ch-PEDOT:PSS 5%, and ch-PEDOT:PSS 10%, respectively.

The morphology of the synthesized beads was investigated through Environmental Scanning Electron Microscopy (ESEM, EVO LS10, Zeiss, Jena, Germany), and only chitosan and ch-PEDOT:PSS 10% beads were analyzed by means of Raman spectroscopy (Labram HR-800 spectrophotometer (Horiba/Jobin Yvon, Kyoto, Japan) with a He−Ne laser source (λ = 632.8 nm), acquisition time 30s, laser power was attenuated to 0.3 mW (10% filter)), and FTIR spectroscopy.

Beads containing only polystyrene sulfonate sodium salt (NaPSS, Sigma Aldrich, St. Louis, Missouri, USA) or Activated Carbon (DARCO, Sigma Aldrich, St. Louis, MO, USA) were prepared for comparison.

Further, 10 mg of NaPSS were dissolved in 9.5 mL of Milli-Q water and the solution was mixed with 100 mg of chitosan and 500 μL of acetic acid. The mixture was left under stirring on a magnetic plate until all chitosan was completely dissolved. The obtained solution was used to obtain chitosan beads containing PSS in the same concentration of ch-PEDOT:PSS 10% beads following the same gelation procedure.

Further, 10 mg of activated carbon was suspended in 10 mL of 5% (*v*/*v*) acetic acid solution and 100 mg of chitosan was added. The mixture was left under stirring on a magnetic plate until all chitosan was completely dissolved. The obtained solution was used to obtain chitosan beads containing AC with a concentration of 1%, following the gelation procedure described above.

### 4.3. Dyes Batch Adsorption Tests

Twenty-three hydrogel beads of chitosan or chitosan-PEDOT (0.1–10% wt) were added to 1 mL of methylene blue (MB) or methyl orange (MO) solutions with a concentration of 10^−5^ M prepared using Milli-Q water (obtained from a Milli-Q Integral 5 system). The pH of the dye solutions after mixing with beads was ~6.2.

The variation in the absorption peak (at 660 nm for MB and 460 nm for MO) was evaluated at regular time intervals (0–24 h) after the addition of hydrogel beads by means of a UV–vis spectrometer (QE 65000 Ocean Optics, Orlando, FL, USA).

The percentage of adsorbed dye was determined using the following equation:

$$\%\;Adsorbed=\frac{A_{0}-A_{t}}{A_{0}}*100$$

where *A*_0_ is the initial value of absorbance of the analyte (proportional to the value of its initial concentration) and *A_t_* is the value of absorbance measured at time *t* after mixing with the adsorbent in the dark. The obtained data were used for the calculation of *q_t_*, which represents the amount of adsorbed pollutant per gram of hydrated adsorbent (mg g^−1^) at any time *t* (min) and for the calculation of *q_e_* (mg g^−1^), which corresponds to the amount of adsorbed pollutant per gram of hydrated adsorbent after the reaching of the equilibrium between the processes of dye adsorption and desorption on the surface of the hydrogel beads under dark conditions and represents the equilibrium adsorption capacity of the tested systems.

The experimental data were fitted with the linear form of the pseudo-second-order model for solid–liquid adsorption according to the following equation:

$$\frac{t}{q_{t}}=\frac{1}{k_{2}q_{e}^{2}}+\frac{t}{q_{e}}$$

where *k* corresponds to the pseudo-second-order kinetic constant.

The performances of the pure chitosan or chitosan-PEDOT hydrogel beads were compared with the adsorption capability of analogous systems made of chitosan and activated carbon (DARCO) or chitosan and PSS, maintaining constant all the experimental parameters (room temperature, sampling times, Milli-Q water, 10^−5^ M dye concentration, no pH adjustment).

In order to obtain more information about the interaction between chitosan and PEDOT with the organic dyes, the solution pH was subsequently buffered at 5.6 using CH_3_COOH and at 9.8–10 using NaOH.

Further adsorption experiments were conducted by preparing dye solutions using not ultrapure water but mineral water (see Table S2 for its chemical analysis).

In these cases, adsorption tests were performed employing as decontaminant only pure chitosan beads and ch-PEDOT:PSS 10% beads.

We tested the adsorption performances of chitosan-PEDOT 10% beads in acidic solutions containing a mixture of MO and MB, both at a concentration equal to 10^−5^ M.

ch-PEDOT:PSS 10% beads were subjected also to reusability tests: after a first adsorption cycle of each single dye lasting 24 h, beads were removed from the dye solution by means of tweezers and resuspended in 1 mL of SDS (sodium dodecyl sulfate) 0.1 M solution for 24 h. At regular intervals, we measured by means of UV–vis spectroscopy the amount of MB or MO extracted from the beads to the SDS solution. After 24h of incubation with the SDS solutions, beads were washed thoroughly with Milli-Q water and resuspended in 1 mL of fresh MB or MO 10^−5^ M solutions. This procedure was repeated to study adsorption performance variations along 5 adsorption cycles.

All adsorption tests were performed on three different bead batches and the data reported in the main text are the average result.

## Data Availability

Data presented in this study are contained within the article and Supplementary Materials.

## Supplementary Materials

The following supporting information can be downloaded at https://www.mdpi.com/article/10.3390/gels10010037/s1, Section S1: FT-IR characterization of extracted chitosan, Section S2. Raman characterization of extracted chitosan, Section S3: Titration of extracted chitosan, Section S4: Spectroscopic characterization of (functionalized) chitosan beads, Section S5: ESEM characterization of (functionalized) chitosan beads, Section S6: Kinetic models for the fitting of the experimental data for adsorption of MB, Section S7: Kinetic models for the fitting of the experimental data for adsorption of MO, Section S8: Comparison with ch-PSS 1% beads, Section S9: Adsorption of MB at different pH values, Section S10: Adsorption of MO at different pH values, Section S11: Adsorption of MB and MO in acidic solution containing both dyes simultaneously, Section S12: Chemical analysis of mineral water used for the preparation of true-to-life dye solutions.
