# Supplementary material for: From Water for Water: PEDOT:PSS-Chitosan Beads for Sustainable Dyes Adsorption
Source: Gels. 2023 Dec 31;10(1):37. doi: 10.3390/gels10010037 (PMC10815287; doi:10.3390/gels10010037)
Supplement: Supplementary file 1 [file gels-10-00037-s001.zip › gels-2775901-supplementary.pdf]

gels

Supporting Information

From Water for Water: PEDOT:PSS-Chitosan Beads for Sustainable Dyes Adsorption

Irene Vassalini [1,2,3,*,†], Marina Maddaloni [2,4,†], Mattia Depedro [1], Alice De Villi [1], Matteo Ferroni [5,6] and Ivano Alessandri [1,2,3]

1 Sustainable Chemistry and Materials Laboratory, Department of Information Engineering, University of Brescia, Via Branze 38, 25123 Brescia, Italy
2 Consorzio Interuniversitario Nazionale per la Scienza e Tecnologia dei Materiali (INSTM), Research Unit of Brescia, Via Branze 38, 25123 Brescia, Italy
3 CNR-INO, Research Unit of Brescia, Via Branze 38, 25123 Brescia, Italy
4 Chemistry for Technology Laboratory, Department of Mechanical and Industrial Engineering, University of Brescia, Via Branze 38, 25123 Brescia, Italy
5 Department of Civil, Environmental, Architectural Engineering and Mathematics, University of Brescia, Via Branze, 43, 25123 Brescia, Italy
6 CNR-IMM Bologna, Via Gobetti 101, 40129 Bologna, Italy
* Correspondence: irene.vassalini@unibs.it
† These authors contributed equally to this work.

Citation: Vassalini, I.; Maddaloni, M.; Depedro, M.; De Villi, A.; Ferroni, M.; Alessandri, I. From Water for Water: PEDOT:PSS-Chitosan Beads for Sustainable Dyes Adsorption. *Gels* **2024**, *10*, 37. https://doi.org/10.3390/gels10010037

Academic Editor: Yi Cao

Received: 30 November 2023
Revised: 25 December 2023
Accepted: 28 December 2023
Published: 31 December 2023

Copyright: © 2023 by the authors. Licensee MDPI, Basel, Switzerland. This article is an open access article distributed under the terms and conditions of the Creative Commons Attribution (CC BY) license (https://creativecommons.org/licenses/by/4.0/).

S1. FT-IR characterization of extracted chitosan

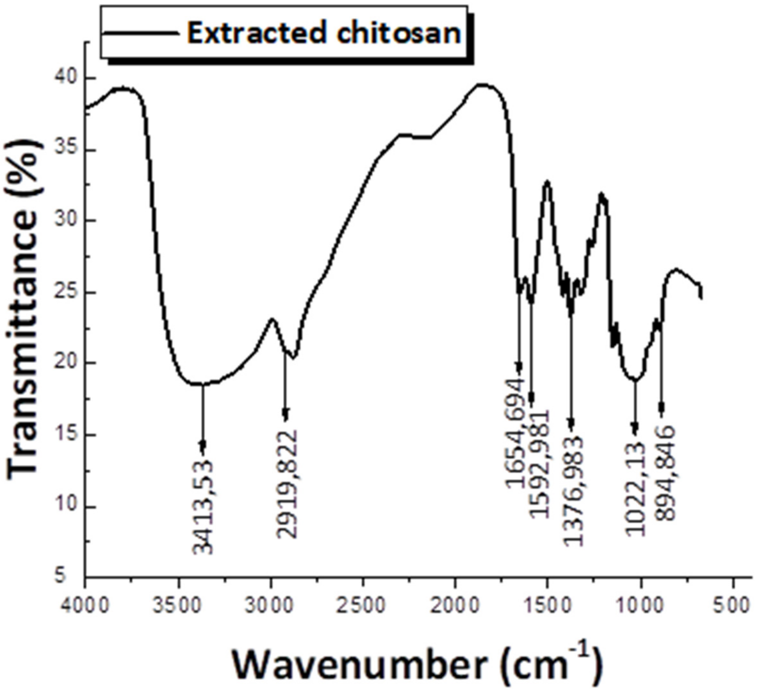

Figure S1. IR spectrum of chitosan powder obtained after the treatment with HCl and NaOH of the household waste shrimp shell and heads.

S2. Raman characterization of extracted chitosan

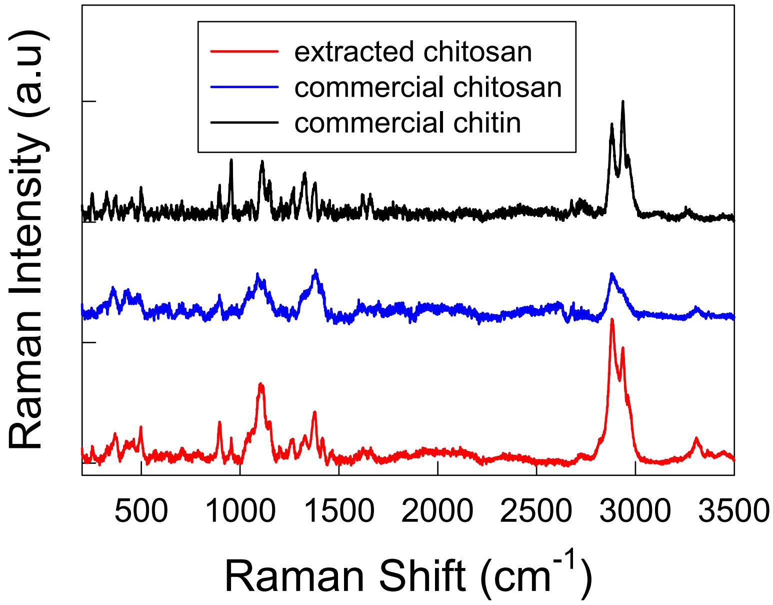

Figure S2. Raman spectrum of chitosan powder obtained after the treatment with HCl and NaOH of the household waste shrimp shell and heads and its comparison with the Raman spectra of commercial chitosan (Biobasic) and commercial chitin (Sigma Aldrich) powders.

S3. Titration of extracted chitosan

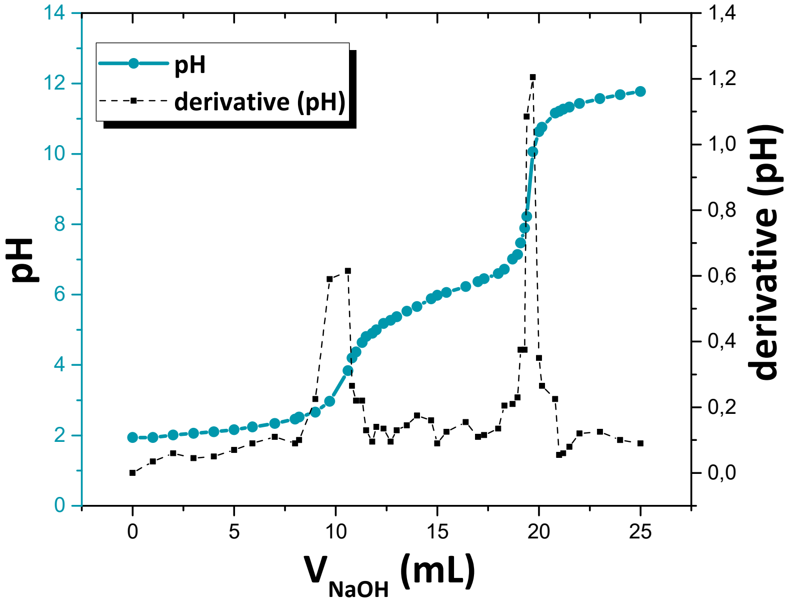

Figure S3. Titration curve of extracted chitosan.

The deacetylation degree (DD%) of extracted chitosan was calculated performing a potentiometric titration: initially chitosan powder was dissolved in an acidic solution (with a final HCl concentration of 0.028 M) and was put in contact with increasing amount of NaOH solution. A curve was created by plotting the variation of the pH as a function of the volume of NaOH 0.1M gradually added. As visible in Figure S3, this curve presents two inflection points, one in correspondence of V_1 and one in correspondence of V_2.

Initial dissolution of chitosan in acidic pH occurs thanks to the protonation of its aminic groups that are de-acetylated, but in these experimental conditions there was an excess of protons and HCl. So, initially, the added NaOH reacted with these free protons, deriving from the excess of HCl, until they are completely neutralized according to the following reaction:

$$\mathbf{HCl}\text{ (excess)} + \text{Na}\mathbf{OH} + \text{Chitosan-NH}_3^+\text{+Cl}^- \rightarrow \text{NaCl} + \mathbf{H_2O} + \text{Chitosan-NH}_3^+\text{+Cl}^-$$

V_1 corresponds to the volume of NaOH that must be added to reach this first neutralization.

When further volume of NaOH was added, also the protons bond to the aminic group of chitosan were neutralized, according to the following reaction:

$$\text{Chitosan-}\mathbf{NH_3}^+\text{+Cl}^- + \text{Na}\mathbf{OH} \rightarrow \text{Chitosan-}\mathbf{NH_2} + \mathbf{H_2O} + \text{NaCl}$$

V_2 corresponds to the volume of NaOH that must be added to complete this second neutralization.

The difference between the two volumes (V_2-V_1) corresponds to the amount of HCl that during the initial dissolution linked to free (de-acetylated) aminic groups of chitosan and allows us to determine the chitosan DD (%). For this calculation we used the formula reported in [1]

$$DD\,\% = \frac{2.03\,(V_2 - V_1)}{m\; + \;0.0042\,(V_2 - V_1)}$$

where *m* corresponds to the mass of the chitosan powder, V_1 and V_2 are the volume of the NaOH added at the two deflection points, *2.03* is a coefficient obtained from the MW of monomeric unit of chitin and *0.0042* is a coefficient obtained from the difference between MW of chitin and chitosan.

In the case reported in Figure S3, V_1= 10.8 mL, V_2= 20 mL, m= 200.3 mg, so that the calculated DD% was equal to 78.2 %.

S4. Spectroscopic characterization of (functionalized) chitosan dried beads

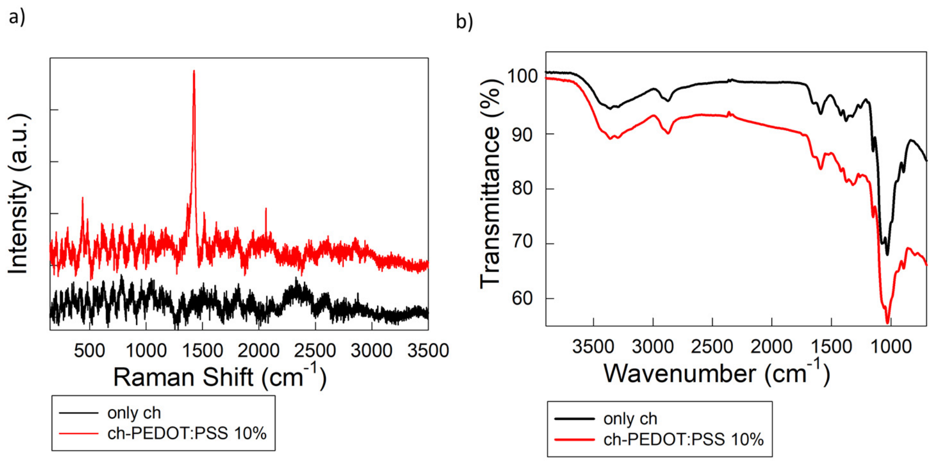

Figure S4. a) Raman spectra of chitosan and chitosan-PEDOT:PSS 10% dried beads; b) FTIR spectra of chitosan and chiotsan-PEDOT:PSS 10% dried beads.

S5. Environmental Scanning Electronic Microspcopy characterization of (functionalized) chitosan beads

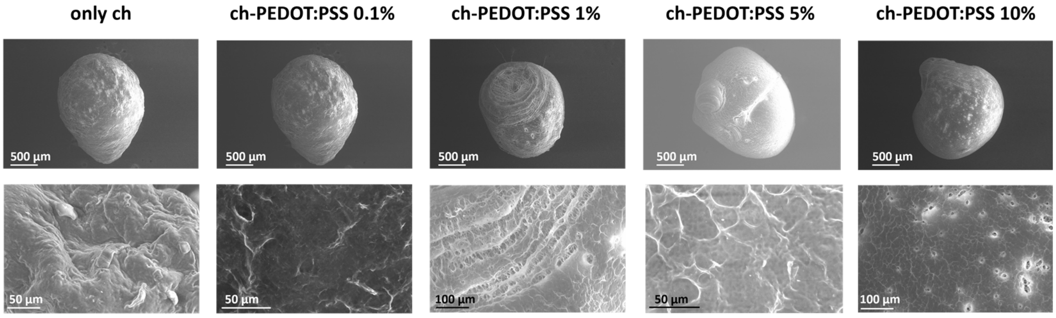

Figure S5. Environmentla Scanning Electrone Microscopy images of chitosan beads containing different percentages of PEDOT:PSS.

S6-S7. Individuation of the best kinetic model for the fitting of adsorption data

From the adsorption data and through the formula

$$q_t = (C_0 - C_t) \times (V/m)$$

where C_0 corresponds to the starting concentration of the dye (mg/L), C_t is the dye concentration at a particular sampling time (mg/L), V is the volume of dye solution (L) and m is the weight of hydrated adsorbent (g), it was possible to calculate the adsorption capacity of the beads at any time (q_t, mg/g) and the equilibrium adsorption capacity (q_e, mg/g).

In order to individuate the best kinetic model for the fitting of the adsorption data, the obtained values of q_t and q_e were used for the creation of the graphs corresponding to the different kinetic models, as reported in **Figure S6** for MB and **Figure S7** for MO.

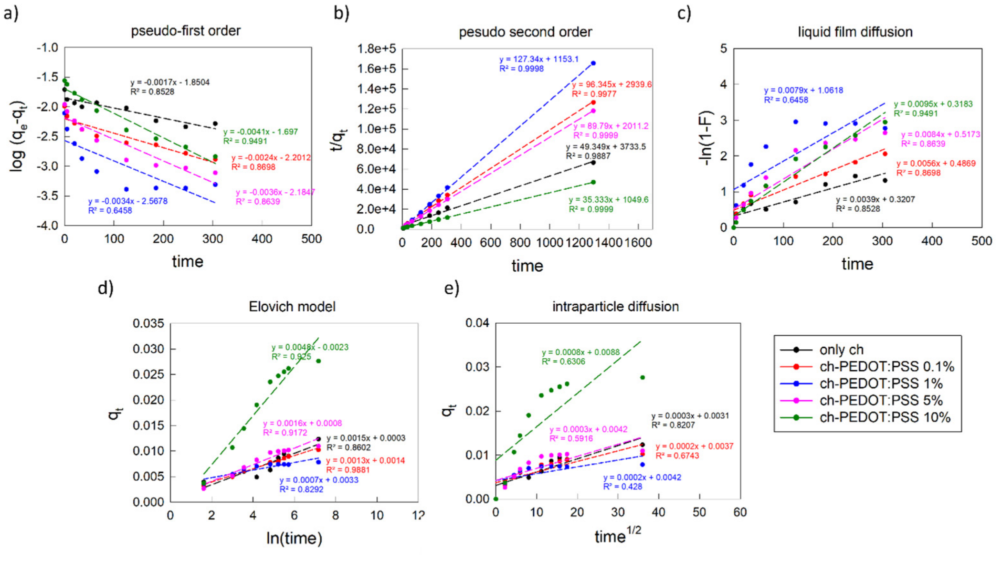

Figure S6. Different kinetic models for the fitting of the experimental data for adsorption of MB. a) pseudo-first order model; b) pseudo-second order model; c) liquid film diffusion model; d) Elovich model; e) intraparticle diffusion model.

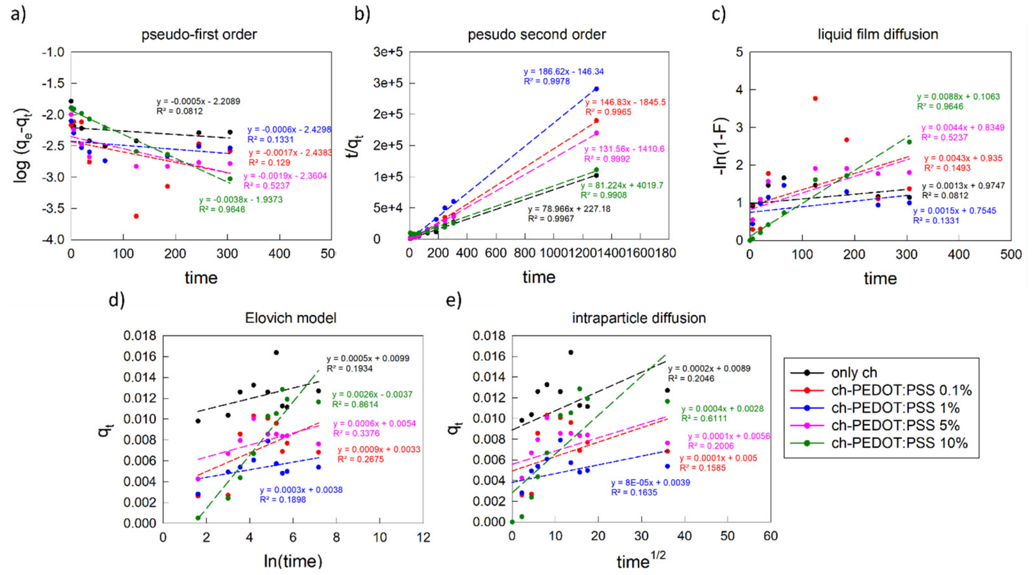

Figure S7. Different kinetic models for the fitting of the experimental data for adsorption of MO.. a) pseudo-first order model; b) pseudo-second order model; c) liquid film diffusion model; d) Elovich model; e) intraparticle diffusion model.

The highest correlation coefficient value ($R^2 > 0.99$) was obtained for the pseudo second order model, whose linear plots are reported also in Figures 2c-d of the main text. According to equation 2

$$\frac{t}{q_t} = \frac{1}{k_2 q_e^2} + \frac{t}{q_e}$$

it was possible to calculate the theoretical value of q_e from the slope of the linear plot of t/q_t versus time (t), as reported in **Table S1.**

Table S1. Comparison between theoretical (obtained from the fitting with the pseudo-second order model) and experimental values of q_e for the different types of chitosan-PEDOT:PSS beads.

	MB 10^{-5}M		MO 10^{-5}M	
	experimental q_e (mg/g)	theoretical q_e (mg/g)	experimental q_e (mg/g)	theoretical q_e (mg/g)
**ch**	0.0123	0.0203	0.0127	0.0127
**ch-PEDOT:PSS 0.1%**	0.0102	0.0104	0.0068	0.0068
**ch-PEDOT:PSS 1%**	0.0078	0.0079	0.0054	0.0054
**ch-PEDOT:PSS 5%**	0.0110	0.0111	0.0076	0.0076
**ch-PEDOT:PSS 10%**	0.0276	0.0283	0.0117	0.0123

S8. Comparison with ch-PSS 1% beads

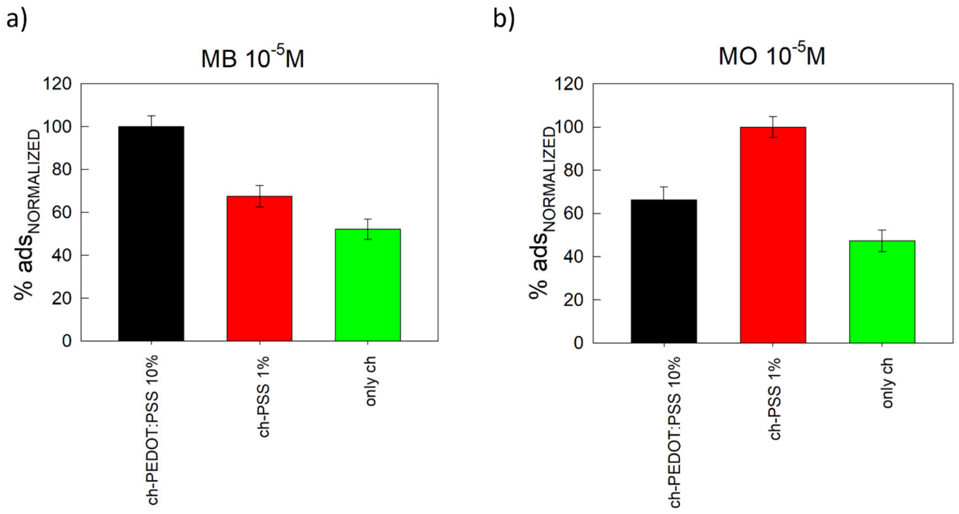

Figure S8. Comparison between the normalized adsorption percentage of only chitosan, ch-PEDOT:PSS10% and ch-PSS1% beads for a) MB 10^{-5} M solution and b) MO 10^{-5} M solution.

S9-S10. Adsorption of MB and MO at different pH values

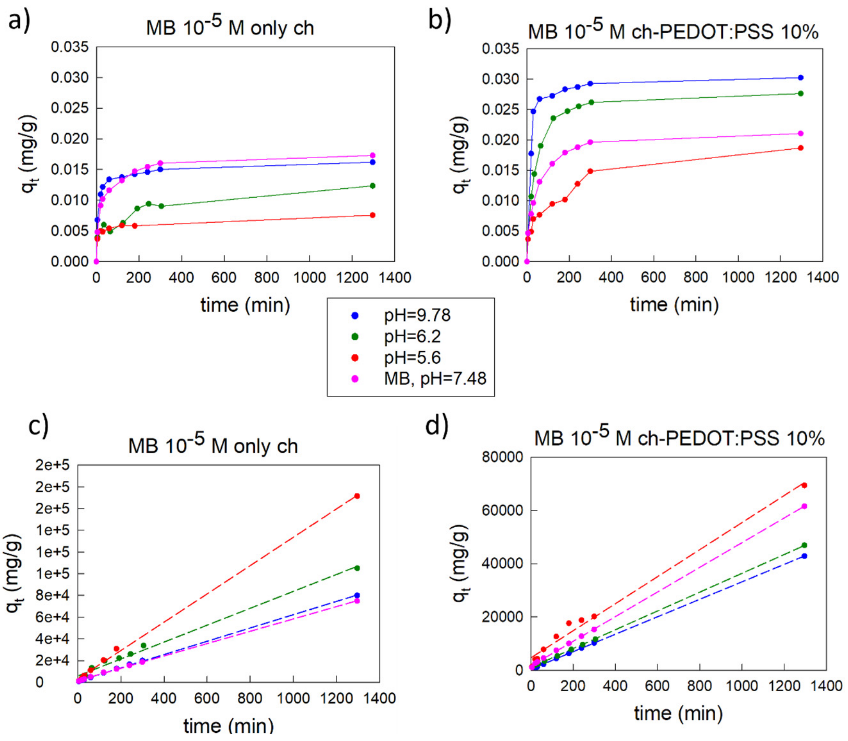

Figure S9. Variation of the adsorption capacity of a) pure chitosan beads and b) chitosan beads functionalized with PEDOT:PSS (10% V) as a function of beads soaking time inside a MB 10^{-5} M solution considering different solution pH values; c, d) corresponding pseudo-second order kinetic models for adsorption of MB in different pH conditions.

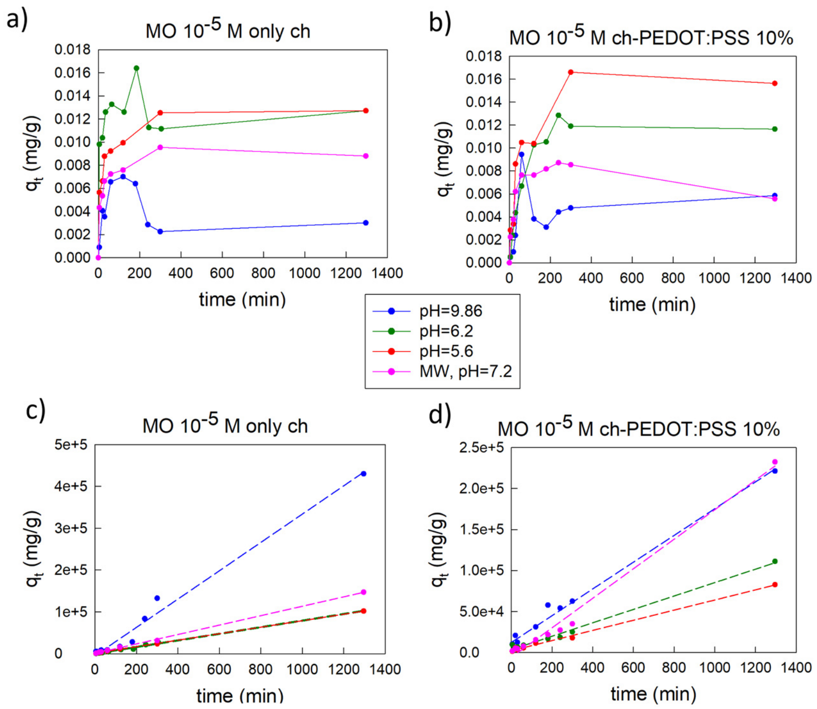

Figure S10. Variation of the adsorption capacity of a) pure chitosan beads and b) chitosan beads functionalized with PEDOT:PSS (10% V) as a function of beads soaking time inside a MO 10^{-5} M solution considering different solution pH values; c, d) corresponding pseudo-second order kinetic models for adsorption of MO in different pH conditions.

S11. Adsorption of MB and MO in acidic solution containing simultaneously both the dyes

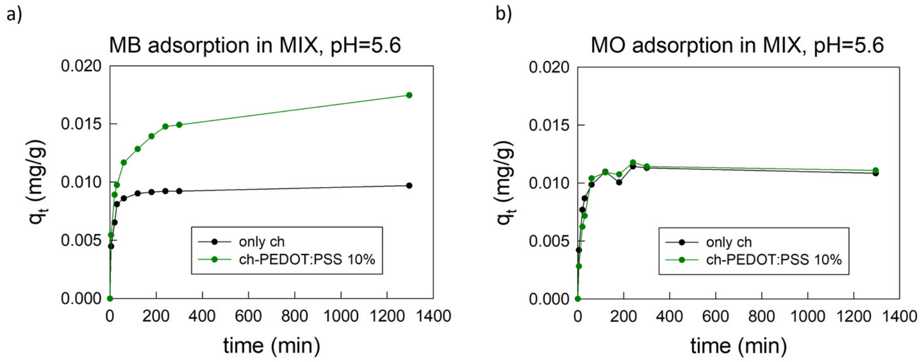

Figure S11. Variation of the adsorption capacity of pure chitosan beads and chitosan beads functionalized with PEDOT:PSS (10% V/V) as a function of beads soaking time inside an acidic solution containing simultaneously MB and MO, both at concentration 10-5M. a) adsorption capacity for MB; b)adsorption capacity for MO.

S12. Chemical Analysis of mineral water used for the preparation of true-to-life dye solutions

Table S2. Chemical composition of bottled water. Chemical-physical constants: Water temperature at the source: 10.8°C · pH at the source: 7.60 · specific electrical conductivity at 20°C: 91 µS/cm · fixed residue at 180°C. 60 mg/L· Hardness °f 5.9 · free CO_2 at the source: 6.0 mg/L.

Substances dissolved in one litre of water expressed in ions			
Ca^{2+}	11.2 mg/L	SO_4^{2-}	5.6 mg/L
Mg^{2+}	3.5 mg/L	NO_3^-	3.8 mg/L
Na^+	2.0 mg/L	Cl^-	2.0 mg/L
K^+	0.70 mg/L	F^-	< 0.1 mg/L
HCO_3^-	50 mg/L	SiO_2	7.1 mg/L

References

1. Varan, N. The Use of Titration Technique and FTIR Bands to Determine the Deacetylation Degree of Chitosan Samples. J. Text. Sci. Eng. **2017**, 07, doi:10.4172/2165-8064.1000288.
